# Analytical validation of the PAM50-based Prosigna Breast Cancer Prognostic Gene Signature Assay and nCounter Analysis System using formalin-fixed paraffin-embedded breast tumor specimens

**DOI:** 10.1186/1471-2407-14-177

**Published:** 2014-03-13

**Authors:** Torsten Nielsen, Brett Wallden, Carl Schaper, Sean Ferree, Shuzhen Liu, Dongxia Gao, Garrett Barry, Naeem Dowidar, Malini Maysuria, James Storhoff

**Affiliations:** 1 British Columbia Cancer Agency, 3427 - 600 W 10TH Avenue, V5Z 4E6 Vancouver, BC, Canada; 2 NanoString Technologies, Inc., 530 Fairview Avenue North, Suite 2000, Seattle, WA, USA; 3 Myraqa, 3 Lagoon Drive, Redwood Shores, CA, USA; 4 Anatomical Pathology JPN 1401, Vancouver Hospital, 855 W. 12th Ave, V5Z 1 M9 Vancouver, BC, Canada

**Keywords:** PAM50, Analytical validation, ROR, Subtype, Breast cancer, Prosigna, NanoString, nCounter, Reproducibility, FFPE, Gene expression

## Abstract

**Background:**

NanoString’s Prosigna™ Breast Cancer Prognostic Gene Signature Assay is based on the PAM50 gene expression signature. The test outputs a risk of recurrence (ROR) score, risk category, and intrinsic subtype (Luminal A/B, HER2-enriched, Basal-like). The studies described here were designed to validate the analytical performance of the test on the nCounter Analysis System across multiple laboratories.

**Methods:**

Analytical precision was measured by testing five breast tumor RNA samples across 3 sites. Reproducibility was measured by testing replicate tissue sections from 43 FFPE breast tumor blocks across 3 sites following independent pathology review at each site. The RNA input range was validated by comparing assay results at the extremes of the specified range to the nominal RNA input level. Interference was evaluated by including non-tumor tissue into the test.

**Results:**

The measured standard deviation (SD) was less than 1 ROR unit within the analytical precision study and the measured total SD was 2.9 ROR units within the reproducibility study. The ROR scores for RNA inputs at the extremes of the range were the same as those at the nominal input level. Assay results were stable in the presence of moderate amounts of surrounding non-tumor tissue (<70% by area).

**Conclusions:**

The analytical performance of NanoString’s Prosigna assay has been validated using FFPE breast tumor specimens across multiple clinical testing laboratories.

## Background

Molecular biomarkers have played an increasingly important role in identifying cancer patients with different prognostic outcomes and in predicting response to chemotherapy [1-3]. Molecular assays targeting these biomarkers are now routinely performed in local pathology labs to help guide treatment decisions in breast cancer [4,5], lung cancer [6], and colorectal cancer [7]. Gene expression analysis has helped identify distinct molecular signatures in breast cancer that have different prognostic outcomes [8-10]. Multigene assays targeting 21 – 70 genes are now routinely used in clinical practice to assess risk of recurrence in early stage breast cancer [11,12], and prospective clinical trials are also underway to provide further supporting evidence for the clinical utility of these assays [13,14]. To date, breast cancer multigene clinical assays have been largely limited to central reference laboratories due to the complexity of performing the test. Ultimately, development of assays with a simplified workflow is required to move these multigene expression tests into the local pathology lab setting, where efficiencies such as shorter turnaround time and direct interaction between laboratory physicians and the clinicians will benefit active patient care.

The PAM50 gene signature measures the expression levels of 50 genes in a surgically resected breast cancer sample to classify a tumor as one of four intrinsic subtypes (Luminal A, Luminal B, HER2-enriched, and Basal-like) [15], which have been shown to be prognostic in both untreated (i.e. no adjuvant systemic therapy) and tamoxifen treated patient populations [15,16]. In addition to identifying a tumor’s intrinsic subtype, the PAM50 signature generates an individualized score estimating a patient’s probability of disease recurrence by weighting the molecular subtype correlations, a subset of proliferation genes, and pathologic tumor size [15,16]. The PAM50 test was adapted to be performed using the nCounter Analysis System in order to develop a simplified workflow that could be performed in a local pathology lab (Prosigna™ Breast Cancer Gene Signature Assay, NanoString Technologies, Seattle). This technology uses multiplexed gene-specific fluorescently-labeled probe pairs [17] to measure gene expression in frozen or formalin-fixed paraffin-embedded (FFPE) tissues with equivalent ease and efficiency [18]. A recent clinical validation performed on RNA extracted from over 1000 FFPE tumor specimens from the ATAC clinical trial demonstrated that the Prosigna risk of recurrence (ROR) score, based on the PAM50 gene expression signature, added significant prognostic information beyond the Oncotype DX® Recurrence Score® in estimating the likelihood of distant recurrence in hormone receptor positive, post-menopausal breast cancer patients [19] treated with endocrine therapy alone. A second clinical validation study performed on over 1400 FFPE patient samples from the ABCSG-8 trial has independently confirmed the clinical validity and demonstrated additional prognostic value in node-positive patients and for the risk of late recurrence [20,21]. Based in part on the results from these clinical studies and the analytical studies described herein, NanoString obtained a CE Mark for its Prosigna assay in 2012, followed by US Food and Drug Administration (FDA) clearance in September of 2013.

Recently, requirements for demonstrating utility of a tumor biomarker were established that include not only clinical validity, but also analytical reproducibility and robustness [22,23]. The results of ATAC and ABCSG-8, including a follow up combined analysis of the two studies [24] meet this high level of evidence (Level I) for clinical validity using archived specimens [22]. The studies described herein were designed to test the analytical validity of decentralized use of the Prosigna assay across multiple clinical testing sites, following established guidelines [25]. These studies were also designed to validate procedures for training laboratory personnel to perform the Prosigna assay on the nCounter system.

## Methods

### NanoString Prosigna assay

The tissue input for the Prosigna assay was FFPE tissue that had been previously diagnosed to contain viable invasive breast carcinoma. The breast tumor tissue must be classified by a pathologist as invasive carcinoma (ductal, lobular, mixed, or no special type). A pathologist reviews an H&E stain of a slide mounted tumor section to identify and circle the region of viable invasive breast carcinoma. The tumor surface area on the H&E stained section must be ≥ 4 mm^2^ per slide, with tumor cellularity ≥ 10%. Non-tumor tissue from outside the circled area is removed by macrodissection of the corresponding unstained slides. RNA was extracted from slide mounted breast tissue sections using a RNA extraction kit manufactured by Roche to NanoString’s specifications [26]. For RNA isolation, a single 10-micron slide mounted tissue section was input for RNA extraction when the tumor surface area measured ≥ 100 mm^2^, whereas 3 slides were input when the tumor surface measured 4-99 mm^2^. Following extraction of total RNA and removal of genomic DNA, RNA was eluted (30 μL volume) and tested to ensure it met the specifications for concentration (≥ 12.5 ng/ μL) and purity (OD 260/280 nm 1.7-2.5).

The NanoString Prosigna assay [26] measures the expression levels of 50 target genes plus eight constitutively expressed normalization genes [15,27,28]. Assay controls are included to ensure that test samples and the test process meet pre-defined quality thresholds. Exogenous probes with no sequence homology to human RNA sequences are included as positive and negative assay controls. Positive controls are comprised of a six point linear titration of *in vitro* transcribed RNA covering an approximately 1000 fold RNA concentration range (0.125 – 128 fM) and corresponding probes [29,30]. Negative controls consist of a set of probes without the corresponding targets. Each assay run includes two reference control samples comprising *in vitro* transcribed RNA of the 58 targets for qualification and normalization purposes.

Extracted RNA samples meeting quality and concentration specifications were hybridized (without reverse transcription or amplification) to capture and reporter probes for the measured genes and assay controls. The multiplexed hybridizations are carried out in a single-tube for 15 – 21 hrs at 65°C using 125 – 500 ng RNA (nominal input of 250 ng). After hybridization, the target-probe complexes were processed on the nCounter Analysis System. Test sample data must meet a minimum threshold for expression of normalizing genes to ensure that the assay signal is high enough for the algorithm to produce precise results. The linearity of the positive control target titration and the non-specific background from negative control probes included in each assay is used to determine whether each assay performed within specification. Since the test is designed to be run in local molecular pathology labs, all quality thresholds are applied automatically to the data by embedded software; any failing metric causes an assay failure notice which prevents output of a Prosigna assay result. For samples meeting all quality thresholds, a clinically validated algorithm is used to determine the intrinsic subtype and ROR score, which are prognostic indicators of risk of distant recurrence of breast cancer [19,21]. The normalized gene expression profile of each breast tumor sample is correlated to prototypical gene expression profiles of the four breast cancer intrinsic subtypes (Luminal A, Luminal B, HER2-enriched, and Basal-like). The primary tumor size (categorical input of ≤ 2 cm or > 2 cm) and normalized gene expression profile of each breast tumor sample is used to calculate the numerical ROR score. Risk categories are assigned to allow interpretation of the ROR score by using pre-specified cutoffs (defined in a clinical validation study) related to risk of distant recurrence after 10 years [19].

Operators for these studies were required to undergo training procedures to demonstrate proficiency, equivalent to what will be used to train users in molecular pathology laboratories for the decentralized test. Each site was given an overview of the NanoString technology and Prosigna assay procedures followed by an in-lab exercise where users were trained and qualified on tissue processing and assay procedures (requiring 10-12 hours of total hands-on time). Briefly, each user extracted RNA from three FFPE breast tumor tissue samples to demonstrate proficiency in tissue processing, and each user processed four prototypical breast tumor RNA samples (one of each intrinsic subtype with known expected ROR score values) along with a negative control sample to demonstrate proficiency on the nCounter Analysis System.

The analytical studies described herein were performed using pre-specified SOPs, statistical analysis plans and acceptance criteria using clinical-grade reagents, instrumentation, and software formatted such that no comparison of results between test centers could even be possible until the study was completed.

### RNA precision: study design

The RNA Precision study assessed the reproducibility of the Prosigna assay using a common template of purified RNA, thereby isolating the device-specific components of analytical validity from variables associated with tissue processing. The experimental design for analytically validating the precision of the assay from RNA was based on Clinical Laboratory and Standards Institute (CLSI) guidelines for the evaluation of precision of *in vitro* diagnostic devices outlined in EP05-A2 [25]. This design measured the variability between and within a number of assay variables including testing site (n = 3), operator (n = 6), reagent lot (n = 3) and assay run (n = 18/site). Two of the three sites used were CLIA-certified, CAP-accredited laboratories at the British Columbia Cancer Agency (Vancouver), and Washington University (St. Louis); the third site was NanoString Technologies (Seattle).

Five pooled breast tumor RNA samples were generated from archived FFPE breast tumor tissue samples containing viable invasive breast carcinoma, to comprise a sample set representing each intrinsic breast cancer subtype and risk classification group (Table 1). Since the samples were pooled breast tumor RNA, a default tumor size category of ≤ 2 cm was used to determine the estimated ROR score, and a default nodal status of node-negative was used to determine risk category. This design ensured that the prototypical gene expression profiles encountered during routine testing were represented within this analytical validation study. Since Luminal subtypes make up the vast majority of the intended use population (hormone receptor positive patients), the study design included three Luminal samples to span the risk classification groups. The identity of each sample aliquot was de-identified using labeled sample tubes with unique, randomly assigned, barcoded IDs to ensure that the operators were blinded to any possible expected results of each test sample.

**Table 1:** RNA precision study sample summary

**Intrinsic subtype**	**Estimated ROR score**	**Risk classification**
Luminal A	30	Low
Luminal B #1	54	Intermediate
Luminal B #2	64	High
Basal-like	55	Intermediate
HER2-enriched	76	High

Molecular characteristics of the five pooled breast tumor RNA samples used in the RNA precision study.

**Figure 1:**
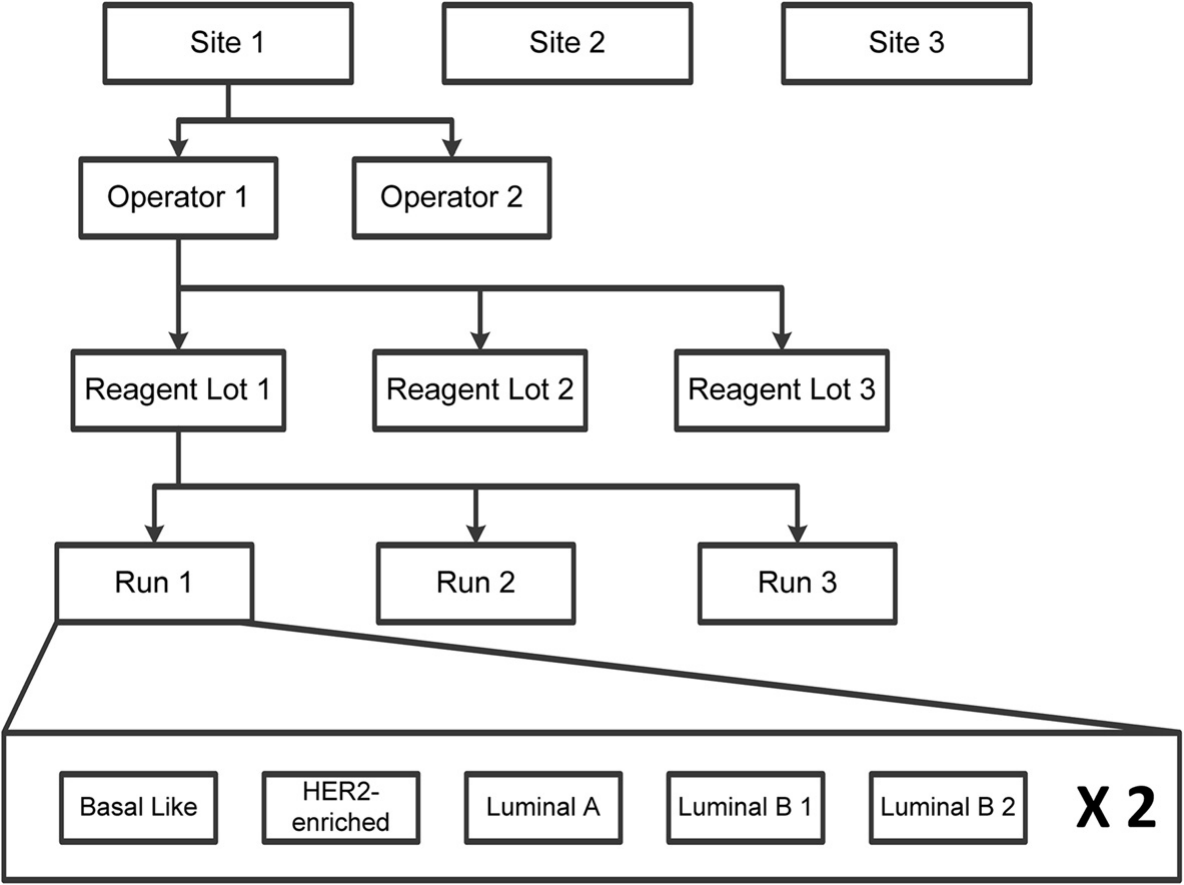
**Overview of the design for the RNA precision validation study.** Five pooled breast tumor RNA samples were tested across several sites, operators, reagent lots, and runs.

Single use aliquots of each pooled breast tumor RNA sample and three reagent lots were distributed to each of the three testing sites to complete the following testing scheme (Figure 1). Each of the five RNA pooled samples was tested in duplicate during each run at the nominal RNA input level for the assay of 250 ng. The positions of the tumor RNA samples within the system (cartridge and strip tube position) were pre-assigned in a randomized and balanced manner for each run. Each operator completed one run on a given day since the assay includes an overnight hybridization step qualifying it as a “long run method” per CLSI EP05-A2. Following a device and study protocol familiarization run, each site completed 18 valid runs (9 by each operator) (Figure 1).

Upon completion of the study the blinded data were collected from each site and merged with the expected test result and study variables (site, operator, reagent lot, etc.) associated with each unique sample ID. The prospectively defined analysis plan was then executed on the merged analysis dataset.

### RNA precision: statistical analysis

The pre-specified primary aim of the RNA precision validation was to demonstrate that there was no significant differences for the continuous ROR score assay output across the three testing sites.

The following variance components model was used to characterize the sources of variability:

ROR Score = site + operator + lot + run + within-run

where all components were treated as random components, and the RNA assay component of variation was defined as the sum of all these components. Variance components were estimated using the R procedure “lmer”. To test whether sites were significantly different, the following versions of the above model were fitted:

ROR Score = site + operator + lot + run + within-run

& ROR Score = operator + lot + run + within-run

where site was now treated as fixed and all other components were treated as random. A likelihood ratio test with 2-degrees of freedom was performed using the fitted models to determine whether the effect of site was significant (α = 0.05). A similar analysis was performed for the assay reagent lots.

For each of the 5 pooled samples, the classifications into the 4 intrinsic subtype categories (Luminal A, Luminal B, Basal-Like, HER2-enriched) were summarized using frequency tables.

### Reproducibility: study design

The reproducibility study assessed the analytical validity of the Prosigna assay, including all steps involving in clinical lab implementation (i.e. tissue handling and RNA isolation SOPs as well as the device-specific assay steps), using a common set of breast cancer tissue samples.

The experimental design for analytically validating the reproducibility from tissue was based on CLSI guidelines for the evaluation of precision of in vitro diagnostic devices outlined in CLSI EP05-A2. This design allows for the measurement of variability between and within a number of assay variables including testing site, FFPE sample block, operator, reagent lot, and assay run.

A set of 43 banked FFPE breast tumor blocks from hormone receptor positive breast cancer patients with confirmed invasive breast carcinoma was selected from the biobank at Washington University at St. Louis for this reproducibility validation study. The sample collection and conduct of this study were conducted in compliance with the study protocols and local IRB procedures. One FFPE block for each case was selected using the following criteria:

1. Every case should represent a unique breast cancer patient

2. All must be primary breast cancers

3. All are pathology confirmed invasive ductal or lobular carcinoma, a mixtures of these types, or classified as no special type

4. All are hormone receptor positive (ER + or PgR+) breast cancer

5. All must have a recorded tumor size

6. FFPE blocks should be < 10 years old

7. A minimum of 10 cases each of ≥ 100 mm^2^ tumor area (1 slide/test) and 4 - 100 mm^2^ tumor area (3 slides/test)

The criterion that at least 10 cases contain ≥ 100 mm^2^ and at least 10 cases contain 4 - 99 mm^2^ tumor area was implemented to validate the number of slides required for the assay. The blocks were not prescreened with the assay prior to inclusion, but it was anticipated that the 43 samples would cover a broad range of ROR scores representative of the intended use population, including both node-negative and node-positive patients, and each risk classification group. Seventeen tissue samples were from node-negative patients, 6 from node-positive patients and 20 were from patients whose regional lymph node status was provided by the biobank as NX.

**Figure 2:**
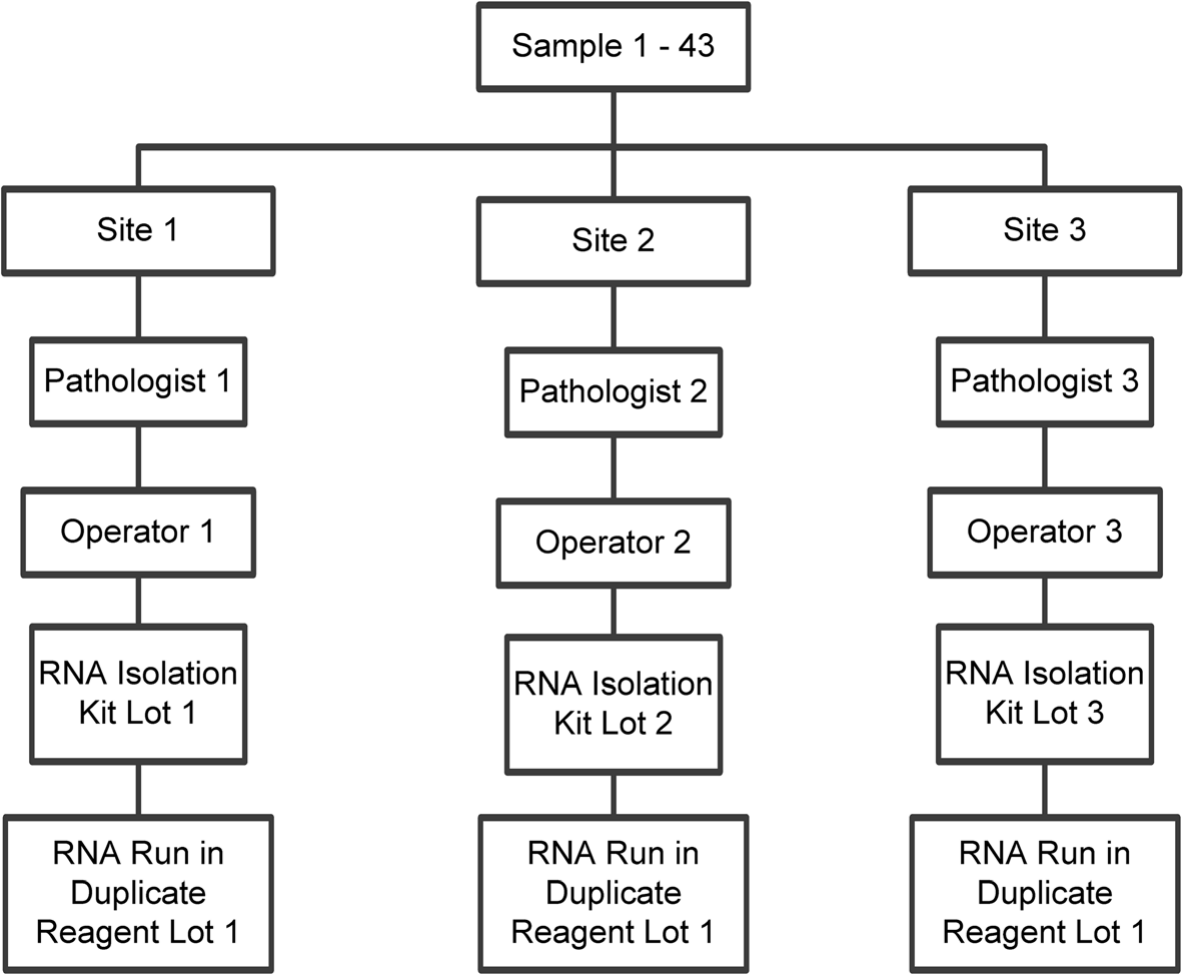
**Overview of the design for the tissue reproducibility validation study.** Tissue samples (1-43) were processed in parallel across different sites, pathologists, operators, and RNA isolation kits.

For reproducibility testing (Figure 2), three sets of serially cut sections, each comprised of one H&E 4-micron stained slide and three 10-micron thick unstained slides, were prepared from each FFPE block. All cut and slide mounted sections were shipped to NanoString and then one set from each of the 43 blocks was distributed to the appropriate testing site for processing. All 43 specimens were reviewed independently by a separate pathologist for each of the three sites.

For each tissue sample, a test run consisting of macrodissection, RNA extraction, and testing with the Prosigna assay was performed by a single operator at each site following the provided standard operating procedures. Each operator performed a minimum of four test runs consisting of up to 10 tissue samples per run. Each batch of tissue samples required a minimum run time of 3 days from tissue processing to result. Isolated RNA that met the quantity and quality specifications from each of the slide mounted sections was tested twice in separate assay runs. Different lots of RNA isolation kit reagents were used at each site, and a single lot of the Prosigna assay kit was used at all three sites.

The test results for all samples remained blinded to all personnel at all sites until the study was complete. Upon completion of the study the blinded Prosigna assay data were collected from each site and merged with the expected test result and study variables (site, operator, reagent lot, etc.) associated with each unique sample ID. The prospectively defined analysis plan was then executed on the merged analysis dataset.

### Reproducibility: statistical analysis

The pre-specified primary aim of the tissue reproducibility validation was to demonstrate the Prosigna assay is highly reproducible, when combining all sources of variation. For this study, “highly reproducible” was defined as a total standard deviation (SD) of less than 4.3 ROR units. The value of <4.3 was chosen because if two samples have true ROR scores that differ by 10 units, a total SD of 4.3 means that 95% of the time the higher of the two will still have a higher individual observed ROR score. A change of 10 ROR units corresponds to an average change in 10-year distant recurrence free survival of 7% and 6% for node negative and node positive patients respectively [19].

The following variance components model was used to characterize the sources of variability:where FFPE Block was treated as a fixed component, and site and section were treated as random components. The “site” term measured the systematic site-specific variation that was constant across all tissue samples (pathologist, technician, extraction kit). The tissue section component measures random variation that differed as a function of review/processing or within FFPE block variation. The error term was derived from the duplicate RNA samples and estimated the combination of run-to-run and within-run variance. Variance components were estimated using the R procedure “lmer”. In the above model, the variance components were estimated from a combined analysis of all FFPE blocks after verifying that were no systematic changes in tissue-specific variation as a function of ROR score.

Measurement = FFPE Block + site + tissue section + error

The tissue and RNA isolation components were estimated using the reproducibility validation and the assay components were estimated using the RNA precision validation. The total variability, $\sigma_{\mathrm{total}}^{2}$, was calculated as:

$$\sigma_{\mathrm{total}}^{2}=\sigma_{\mathrm{tissue}}^{2}+\sigma_{\mathrm{RNA}\;\mathrm{assay}}^{2}$$

where $\sigma_{\mathrm{tissue}}^{2}$ was estimated as the sum of the site-to-site and section component estimated in the tissue reproducibility study, and $\sigma_{\mathrm{RNA}\;\mathrm{assay}}^{2}$ was estimated as the total variation from the RNA precision study.

Additional categorical analyses were performed using two classifications:

•3 risk-categories (low, intermediate, and high) using both the node-negative and node-positive cutoffs,

•4 intrinsic subtype categories (Luminal A, Luminal B, Basal-Like, HER2-enriched)

RNA from each tissue sample was tested twice at each site so there are 4 possible comparisons between sites for each tissue sample leading to a total number of possible comparisons of 4*number of tissue samples. For each of the two classification schemes (risk category or subtype), the pair-wise concordance between sites was estimated as the fraction of all possible comparisons that were concordant and an exact-type 95% confidence interval was calculated.

In addition, a post hoc analysis compared the normalized gene expression from the 50 classifier genes between the tissue replicates from all valid specimens tested at each site using a linear regression and correlation analysis

### RNA input: study design

**Figure 3:**
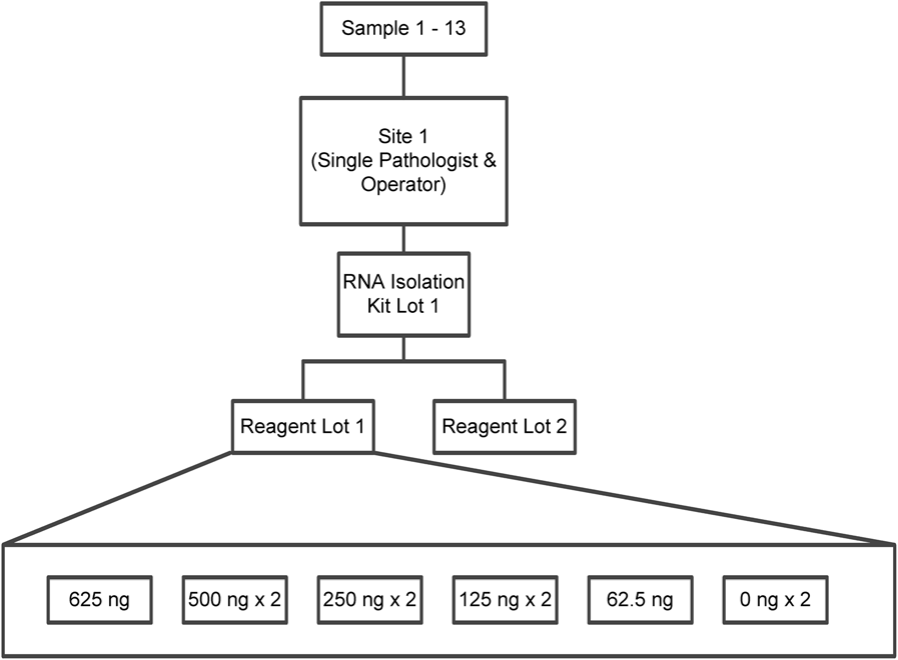
**Overview of the design for the RNA input study.** RNA from 13 tissue samples was tested across and beyond the RNA input range specified for the assay.

Thirteen FFPE breast tumor blocks containing pathologically-confirmed infiltrating ductal carcinoma were obtained and RNA was extracted from multiple slide mounted tissue sections from each block using the defined procedure (Figure 3). The individual RNA isolates from each FFPE block were pooled. Each pooled tumor RNA sample was tested in duplicate across three RNA input levels within the assay specification range (500, 250, and 125 ng) and in singlet at two additional RNA input levels outside of the specification range (625, 62.5 ng). Two no-target (water) measurements were also tested in duplicate on every run. All tumor RNA samples were assumed to be node-negative with a tumor size of ≤ 2 cm for this analytical study since these clinical covariates have no impact on the measured variation in the ROR score. All samples were tested using two different Prosigna assay reagent lots.

### RNA input: statistical analysis

The pre-specified primary aim of the RNA input study was to demonstrate the Prosigna assay results were unchanged at the extremes of the assay specification range (125 and 500 ng RNA) regardless of the assay reagent kit lot used. For each kit lot, the test statistic was the average difference between the mean ROR score at a given input level $\left({\overset{¯}{\mathrm{ROR}}}_{\mathrm{Lj}}\right)$ and the mean ROR score at the nominal level $\left({\overset{¯}{\mathrm{ROR}}}_{\mathrm{Nj}}\right)$:

$$\mathrm{Average}\;\mathrm{Difference}=\frac{1}{n}\overset{n}{\underset{j=1}{\sum }}\left({\overset{¯}{\mathrm{ROR}}}_{\mathrm{Lj}}-{\overset{¯}{\mathrm{ROR}}}_{\mathrm{Nj}}\right)$$

where the average is across the n different samples. In this equation, ${\overset{¯}{\mathrm{ROR}}}_{\mathrm{Nj}}$ is the average of two replicates at the nominal level and ${\overset{¯}{\mathrm{ROR}}}_{\mathrm{Lj}}$ s the average of two replicates for input levels within specification, or is the single result for input levels outside of specification. Equivalence was pre-defined as an observed absolute average ROR difference significantly less than 3. To test the non-equivalence hypothesis that the true absolute mean difference is greater than 3, a 90% confidence interval for the difference was calculated. This 90% confidence interval corresponds to the two one-sided test approach for bioequivalence [31]. The input level was determined to be equivalent to the nominal level if the 90% confidence interval is completely contained within -3 and 3.

For each pooled sample a linear regression and correlation analysis was also performed between each replicate at each RNA input level and one of the two replicates run at 250 ng of RNA. The difference in the ROR score (ΔROR) from the nominal RNA input level (250 ng) for each replicate at each RNA input level was calculated by subtracting the ROR score calculated from one of the two replicates run at 250 ng from ROR scores calculated at the other input levels. Additionally, the ΔROR was calculated and linear regression and correlation analyses were also performed between the two replicates at 250 ng. The mean ΔROR, slope, intercept, and correlation values (with 95% confidence intervals) were calculated using the pairwise comparisons for all passing samples at each input level for both kit lots.

For the no-target (water) samples, the percentage of samples failing the minimum threshold for expression of normalizing genes was calculated. All no-target samples were required to give a failing test result.

### Tissue interferents: study design and analysis

Twenty three FFPE breast tumor blocks were obtained containing pathologically-confirmed infiltrating ductal carcinoma microscopically-assessed to have 10 – 95% of the total tissue area containing normal/non-tumor tissue. Pathologists identified additional tumor interferents (DCIS, necrotic tissue, or blood/hemorrhagic tissue) within or near the margins of the tumor in ten of the 23 blocks.

For each FFPE breast tumor block, H&E stained slides were prepared and up to nine unstained sections were cut and mounted on slides. For the inclusion of the interferent, the sections were processed according to the assay procedure with the exception that identified normal/non-tumor tissue or any additional interferents were included in the isolation (“non-macrodissected slides”). For the macrodissection where the non-tumor and other interferents were removed, three or (in the case of small tumor surface areas) three and six slides were processed according to the Prosigna assay protocol.

**Figure 4:**
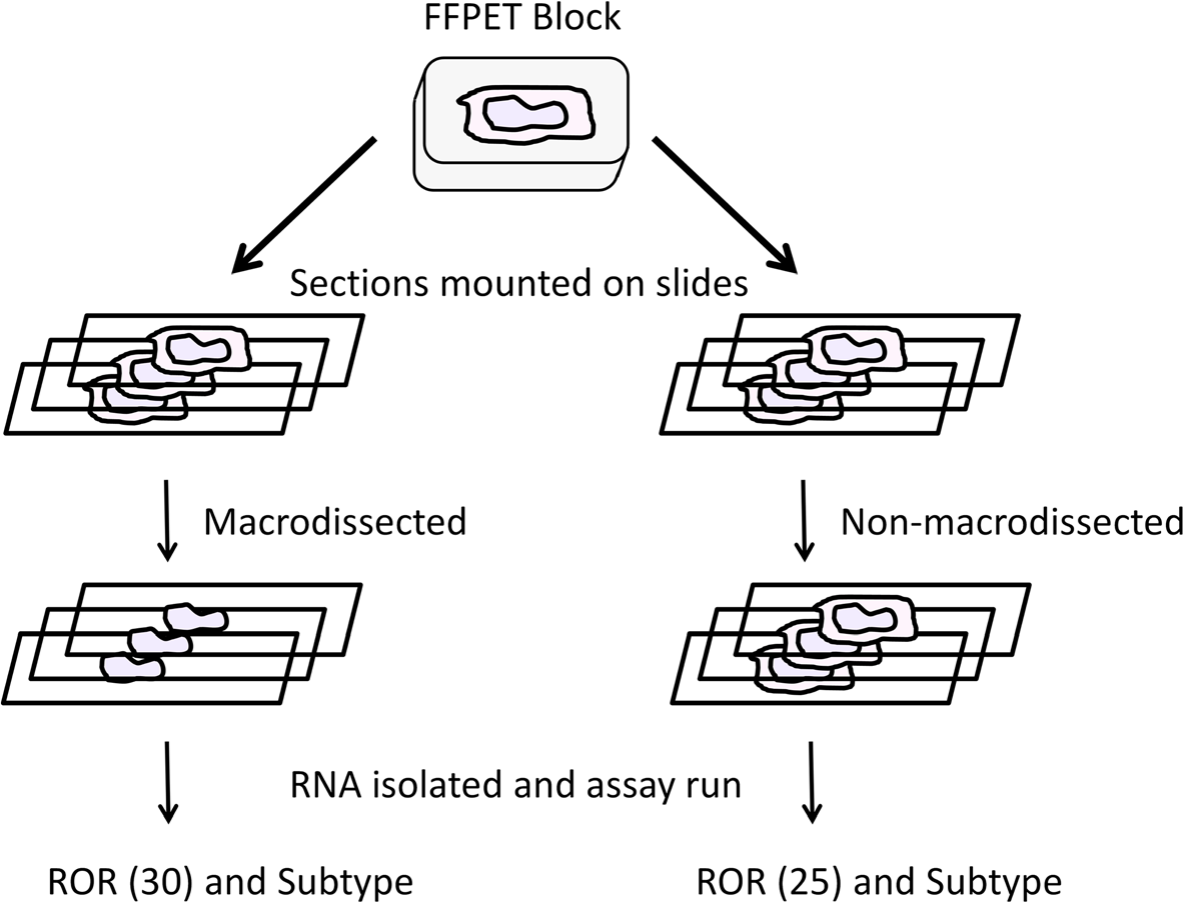
**Overview of tissue processing for assessing the effect of tissue interferents.** Multiple sections from FFPE breast tumor blocks were mounted onto slides and processed with or without macrodissection. The change in ROR score (ΔROR) is calculated as the ROR score from the non-macrodissected slides minus the ROR score from the macrodissected slides (or in the illustration ΔROR = 25 – 30 = -5).

The change in ROR (ΔROR) due to the interferent was calculated using the ROR score from the non-macrodissected slides minus the ROR score from the macrodissected slides (Figure 4). For the tissue blocks where three and six macrodissected slides were independently isolated and both produced a passing assay result, the average of the two ROR scores were used to calculate the ΔROR.

## Results

### RNA precision: variance components analysis

The precision of the Prosigna assay starting from RNA was assessed with 5 pooled breast tumor RNA samples each tested 36 times at each of the three sites. There were no individual test samples that failed the pre-specified data QC metrics in the software so the analysis includes 540 results from 54 valid runs. For all five tumor RNA samples, the total SD was less than 1 ROR unit on a 0 - 100 scale (Table 2), and there was 100% concordance between measured subtype result and expected subtype result as well as measured and expected risk group. More than 60% of the measured variability came from within-run variance (repeatability) while less than 2% of the variance was attributable to site-to-site variance or operator-to-operator variance. The differences in mean ROR scores between sites were less than 0.5 ROR units on a 0-100 scale and were insignificant for all tested samples (Additional file 1: Table S1). The contribution to overall variance by the three reagent lots was approximately 20% of the total variance on average, but the differences were all less than 1 ROR unit. At each site, the normalized gene expression between RNA replicates was highly correlated with slopes ranging from 0.98 – 1.00, intercepts at 0, and r values of 0.99.

**Table 2:** Variance components for the five pooled RNA samples across 108 replicates

**Pooled RNA sample**	**Mean ROR score**	**Variance component (%)**					**Total variance**	**Total SD**
		**Reagent lot**	**Site**	**Operator**	**Run**	**Within-run**		
Basal-like	55.4	0.059 (20%)	0.000 (0%)	0.000 (0%)	0.046 (15%)	0.194 (65%)	0.299 (100%)	0.55
HER2-enriched	76.2	0.165 (37%)	0.000 (0%)	0.000 (0%)	0.000 (0%)	0.277 (63%)	0.442 (100%)	0.66
Luminal A	31.4	0.010 (2%)	0.000 (0%)	0.000 (0%)	0.134 (30%)	0.296 (67%)	0.44 (100%)	0.66
Luminal B 1	55.0	0.105 (18%)	0.000 (0%)	0.000 (0%)	0.046 (8%)	0.426 (74%)	0.576 (100%)	0.76
Luminal B 2	64.8	0.119 (21%)	0.014 (2%)	0.000 (0%)	0.064 (11%)	0.380 (66%)	0.576 (100%)	0.76

The percent of total variance is listed below the estimated variance.

**Figure 5:**
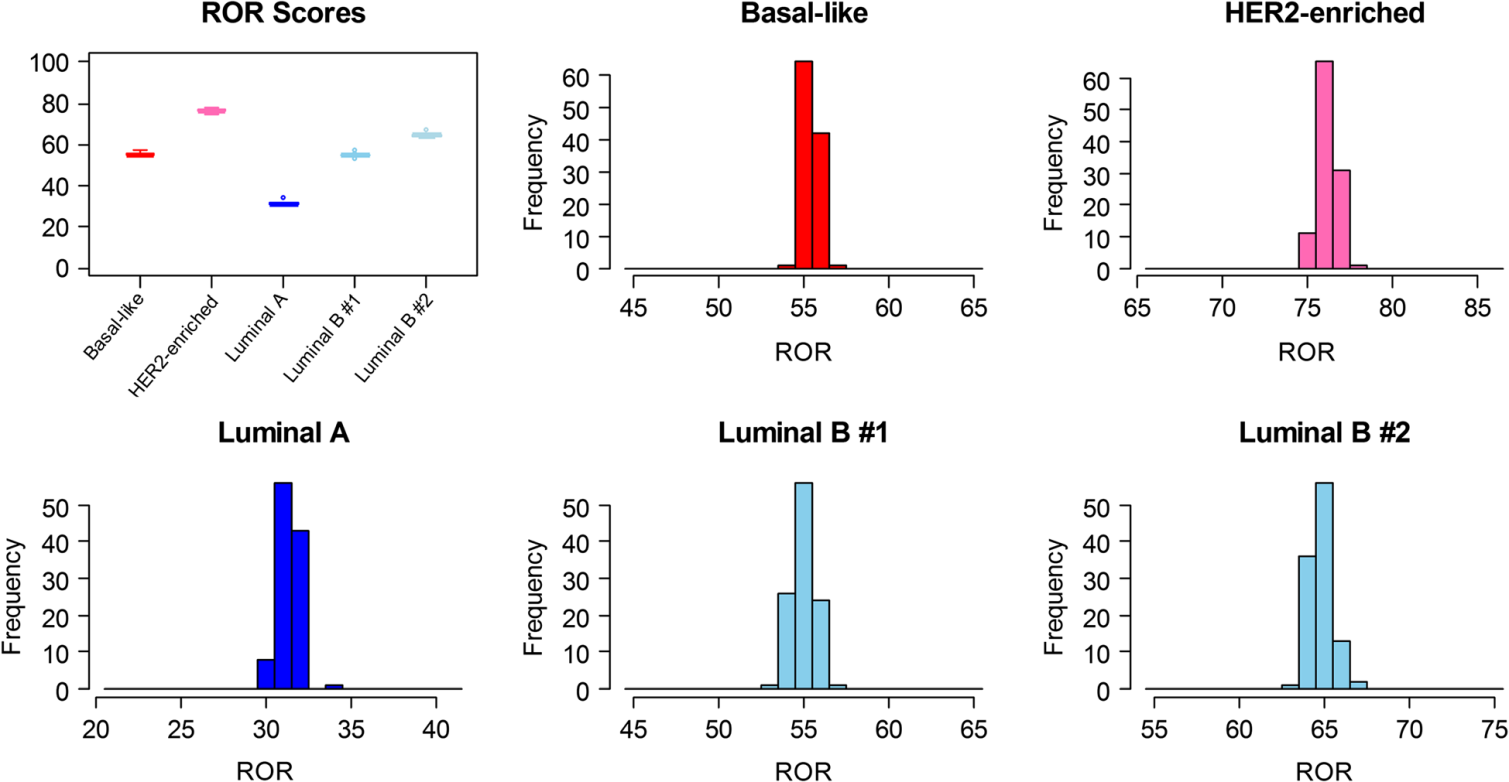
**Distribution of 108 ROR scores measured for each of the 5 Pooled RNA samples.** Boxplots show the distribution of ROR scores relative to the 0-100 range and the histograms show the frequency of the measured ROR scores on a 20-point range. Boxplots and histograms are colored by the intrinsic subtype result for each sample.

The distribution of measured ROR scores for each of the five pooled RNA samples was also examined across the three lots, six users and three test sites. The range of ROR scores for the 108 independent measurements was ≤4 units for each of the 5 sample pools (Figure 5).

### Reproducibility: test sample quality control and characterization

The call rate for the 43 tissue samples evaluated was 95%, 93%, and 100% for sites 1, 2, and 3 respectively. Forty samples yielded results at all sites (RNA isolation of one sample at one site required repeating). One tissue sample yielded results at 2 sites, and 2 samples yielded results at a single site, while the other sites did not obtain sufficient RNA to perform the assay for these samples. The measured tumor surface area for 4/5 RNA isolation failures was very small (≤ 15 mm^2^). One hundred percent (100%) of samples passing tissue review and RNA isolation specifications yielded passing results from the Prosigna assay.

**Figure 6:**
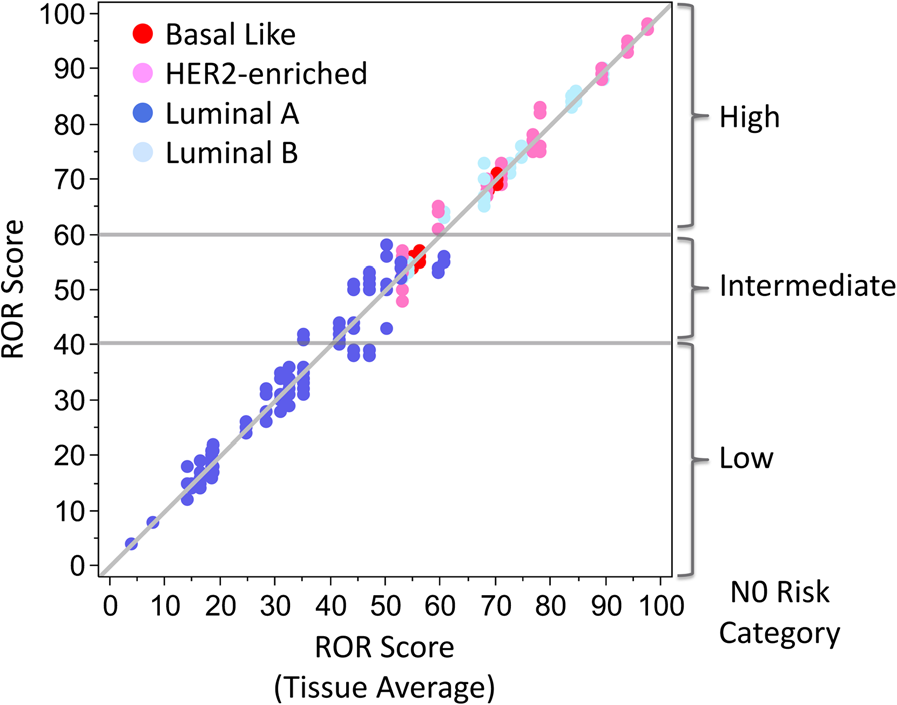
**Reproducibility of the ROR score in the tissue reproducibility study.** Average tissue block ROR compared to the individual ROR score for all samples. Data are colored by the intrinsic subtype result. The high, intermediate, and low node negative risk categories are shown to the right of the figure with the risk thresholds shown as lines in the body of the figure.

The calculated test results from the 43 tissues across all sites represent a wide range (94 units) of ROR scores (Figure 6) and all risk categories when applying the node-negative or node-positive ROR score cutoffs to all samples. All four intrinsic subtypes were also represented among the 43 specimens. The two samples where RNA could only be successfully isolated at one site were excluded from all subsequent statistical analysis as there was no available data for comparing across sites. Both of these samples had ROR scores of less than 10 and were classified as Luminal A.

### Reproducibility: variance components analysis (primary objective)

Table 3 shows the results of the variance components analysis using all 41 tissue specimens where replicate measurements were available. The “tissue section” variation, which consists of variation contributed by within FFPE block sections, pathology review, and tissue processing, was the dominant source of variation (> 90% of total variance). The differences on average between the sites were negligible (< 1% of total variance). The combined run-to-run variability and within-run variability in the assay (determined from the duplicate measurements from each RNA isolation from the reproducibility study) was consistent with the variability measured in the RNA-precision study (variance of 0.51 compared to 0.47 for the RNA-precision study).

**Table 3:** Total variability (from tissue and RNA processing) of the Prosigna assay

**Tissue processing variability**		**RNA processing variability**	**Total variability**	**Total SD**
**Site**	**Within block/process**			
0.10	7.72	0.47	8.29	2.9

The total SD of 2.9 is on a 0-100 ROR scale.

The total SD including all source of variation (tissue and RNA processing variability) was 2.9 indicating that the Prosigna assay can measure a difference between two ROR scores of 6.75 with 95% confidence.

### Reproducibility: subtype and risk category classifications concordance

The site-to-site concordances for the two categorical classifications are shown in Table 4, in each case with exact-type 95% confidence intervals. For each comparison (subtype and node negative and positive risk categories), the average concordance between sites was at least 90%. There were no samples where the risk category changed from low risk to high risk (or vice versa) between or within sites when the samples were assumed to be from node negative patients. There were only two intermediate/high risk samples that did not give identical subtypes across all 6 replicates:

•One sample had duplicate Luminal A results at one site and duplicate Luminal B results at each of the other two sites.

•One specimen had duplicate Luminal A results at one site, duplicate HER2-enriched results at another site and one each of Luminal A and HER2-enriched at the third site.

**Table 4:** Concordance of subtype calls and risk categories between the three sites

**Comparison type**	**Pairwise Concordance [95% CI]**			**Average concordance**
	**Site 1 vs. Site 2**	**Site 1 vs. Site 3**	**Site 2 vs. Site 3**	
	**(n = 40)**	**(n = 41)**	**(n = 40)**	
Subtype	96.3%	98.8%	95%	97%
	[86.4%–99.5%]	[91.0%–100%]	[83.1%–99.3%]	
Risk Category	87.5%	92.7%	90%	90%
(Node Negative)	[73.2%–95.8%]	[80.1%–98.4%]	[76.4%–97.2%]	
Risk Category	90.0%	95.1%	95.0%	93%
(Node Positive)	[76.9%–96.0%]	[83.9%–98.7%]	[83.5%–98.6%]	

The pairwise (site to site) concordance is reported with the 95% confidence interval.

### Reproducibility: pairwise correlation coefficients of gene expression

The average intercept, slope, and Pearson’s correlation of the pair-wise comparisons between sites are reported with the 95% confidence interval (Table 5). The gene expression between tissue replicates was highly correlated between sites with slopes ranging from 0.97 – 1.00, intercepts at 0, and r values of 0.98 or greater. Equivalent or higher correlation values were observed when a similar analysis was performed for the RNA replicates tested at each site (Additional file 2: Table S2). Additionally, hierarchical clustering analysis demonstrated that tissue sample and RNA sample replicates were always and only clustered together across a wide range of expression in each of the 50 genes across all samples tested (Additional file 3: Figure S1).

**Table 5:** Site to site gene expression comparisons from the tissue reproducibility study

**Comparison**	**Pairwise (n)**	**Intercept**	**Slope**	**Pearson**
		**[95% CI]**	**[95% CI]**	**[95% CI]**
All Sites	121	0.00	0.98	0.98
		[-0.01–0.01]	[0.97–0.99]	[0.98–0.98]
Site 1 vs. Site 2	40	0.00	0.97	0.98
		[-0.01–0.01]	[0.95–0.98]	[0.97–0.98]
Site 1 vs. Site 3	40	0.01	1.00	0.98
		[0–0.02]	[0.98–1.01]	[0.98–0.99]
Site 2 vs. Site 3	41	-0.01	0.99	0.99
		[-0.02–0]	[0.97–1]	[0.98–0.99]

Pairwise correlations, slopes, and intercepts of normalized 50 genes for tissues replicates from the tissue reproducibility study. The average intercept, slope, and Pearson’s correlation of the pair-wise comparisons are reported with their 95% confidence intervals.

### RNA input: test sample quality control

The average ROR score for the tested samples covered a broad range (20 – 82) and all intrinsic subtypes – including 5 Luminal A, 4 Luminal B, 3 HER2-enriched and 1 Basal-like sample (Additional file 4: FigureS2). One FFPE block was tested with a single kit lot due to insufficient RNA mass from the isolation for the second lot. Two runs (each with different samples) failed to provide passing results for one of the two lots tested due to a processing error detected by system controls with insufficient RNA to repeat the assay. All measured no-target samples (n = 46) were well below the threshold for signal and yielded a failing test result (0% call rate). All tumor RNA measurements within assay specification (n = 138) yielded a passing test result (100% call rate). One hundred percent (100%) of specimens with input above specification (625 ng) yielded a passing test result. Eighty-three percent (83%) of specimens (10/12) tested at input below specification (62.5 ng) yielded a test result in lot 1, as did 100% in lot 2.

### RNA input: ROR score difference and pairwise correlation coefficients of gene expression

For each of the two reagent lots tested, the confidence interval around the mean ROR score difference between the nominal input and the RNA input limits (125 and 500 ng) were completely contained within -3 and 3 ROR units. The ROR scores at 125 and 500 ng RNA were therefore equivalent to those at the target input concentration of 250 ng for each of the two reagent kit lots tested meeting the primary objective of the study. Of note, when characterizing the RNA levels outside of the assay specification, the ROR scores at 62.5 ng RNA were not equivalent (with an upper confidence limit at 3.26) to those at the target input concentration of 250 ng for one of the two lots tested. This illustrates the importance of performing the assay according to the defined procedure.

When the lots were combined the normalized gene expression values and ROR scores were consistent to those at the target input concentration of 250 ng within and even outside the RNA input limit specifications (Table 6). Characterization of intrinsic subtype across the samples tested shows a 100% concordance in subtype call across all samples and inputs. Similarly, there is a 100% concordance by risk classification across all samples and inputs.

**Table 6:** Comparison of gene expression at different masses from the RNA input study

**Mass (ng)**	**Pairwise (n)**	**Pearson**	**Slope**	**Intercept**	Δ**ROR**
		**[95% CI]**	**[95% CI]**	**[95% CI]**	**[95% CI]**
62.5	21	0.97 [0.93–0.99]	0.96 [0.91–1.00]	-0.02 [-0.05–0.01]	0.48 [-1.27–2.22]
125	46	0.99 [0.97–0.99]	0.98 [0.96–1.01]	-0.01 [-0.03–0.01]	-0.04 [-0.89–0.8]
250	23	0.99 [0.98–1.00]	1.00 [0.99–1.01]	0.00 [-0.01–0.01]	-0.39 [-0.96–0.17]
500	46	0.99 [0.99–1.00]	0.97 [0.96–0.99]	0.02 [0.01–0.04]	-0.57 [-1.39–0.26]
625	23	0.99 [0.98–1.00]	0.95 [0.92–0.99]	0.03 [0.01–0.06]	-0.78 [-2.2–0.63]

Pairwise correlations, slopes, and intercepts of normalized 50 genes and change in ROR score for replicate RNA Hybridizations with different mass inputs. The average intercept, slope, Pearson’s correlation, and change in ROR for the pair-wise comparisons are reported with their 95% confidence intervals.

### Tissue interferents: test sample quality control

Out of 23 samples six were Luminal A, seven were Luminal B, two were HER2-enriched, and eight were Basal-like. The average ROR score for the 23 samples covered a broad range (10 – 83), (Additional file 5: Figure S3).

### Tissue interferents: impact on ROR score

**Figure 7:**
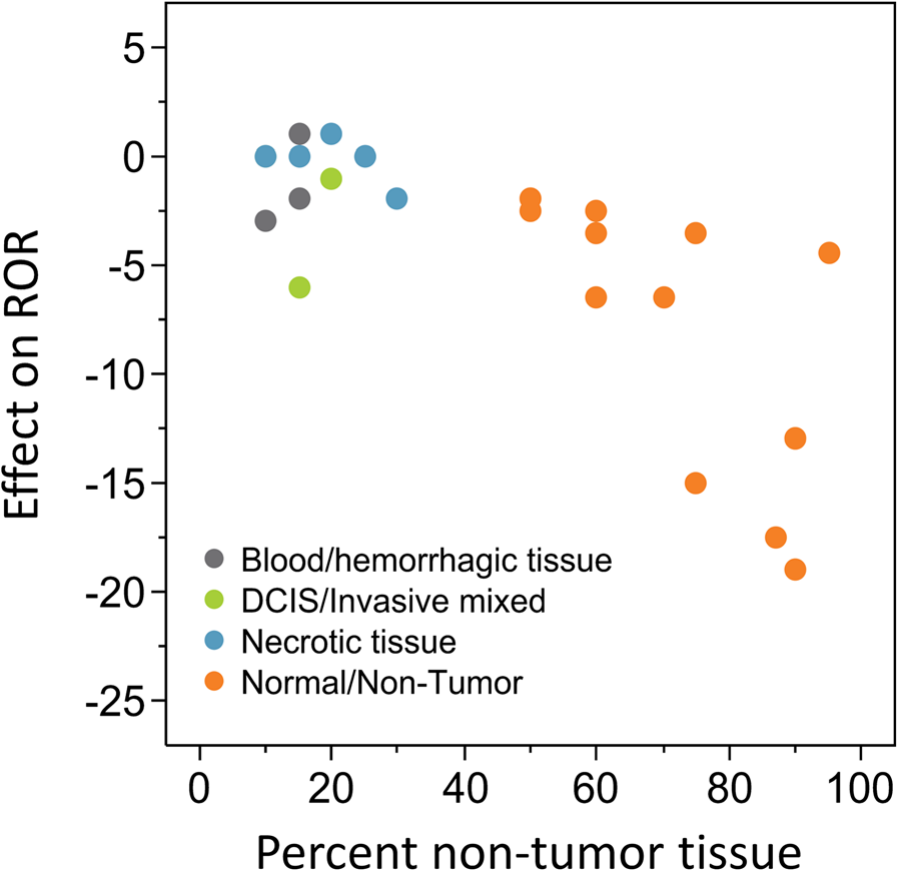
**Effect of non-tumor tissue on the ROR score.** The impact of including adjacent non-tumor tissue on ROR was assessed by determining the change in test results from slide mounted sections with vs. without macrodissection of adjacent non-tumor tissue. Data colors represent if the interferent was only normal/non-tumor tissue or if additional non-tumor interferents (DCIS, necrotic tissue, or blood/hemorrhagic tissue) were identified within or near the margins of the tumor.

As the amount of adjacent non-tumor tissue increases there is an increasing risk that the reported ROR score will be an underestimate or negatively biased (up to -19 ROR score units for samples containing 95% non-tumor tissue) estimate of a patient’s risk of recurrence (Figure 7). Elimination of the macrodissection step required by the assay also caused a change in subtype determination for five out of 23 samples. Three Luminal B samples, one HER2-enriched, and one basal-like sample were classified as Luminal A due to inclusion of adjacent non-tumor tissue. In contrast, the presence of intratumor hemorrhage, necrosis or DCIS (not removed by macrodissection) had little effect on ROR.

## Discussion

Breast cancer gene expression testing has been the subject of many studies demonstrating its capacity to stratify breast cancers by prognostic risk [9,15,16,32,33]. Increasingly, studies are also showing the value of such signatures to predict response to therapy, for example by using these tests to evaluate archival specimens from randomized clinical trials [34-36]. The integration of molecular genomic testing into cancer care is an active area of development, with huge genomic datasets becoming available. Great improvements in experimental design and bioinformatic analysis have led to the development of robust signatures ripe for translation into clinical tests. Studies applying these signatures to different clinical series with observational, case-control, cohort and randomized trial designs have generated increasingly strong evidence for clinical validity, particularly in breast cancer [19,34,35,37]. It is in this backdrop that the Evaluation of Genomic Applications in Practice and Prevention (EGAPP) working group was formed to guide best practices in experimental design and the interpretation of evidence for utility in clinical practice [23]. Fundamental to EGAPP criteria is the concept that clinical utility requires not only clinical validity (linking test results to clinical presentation, treatment and outcome), but just as importantly, analytical validity (the capacity of the test classifier to be sensitive, specific and reproducible in practice). However, EGAPP found that relatively few studies of breast cancer molecular classifiers have directly reported on analytical reproducibility [38].

Analytical reproducibility is a requirement for the implementation of all diagnostic tests, but it is especially critical for decentralized tests given the challenges of maintaining reproducibility across pathologists, technical operators, and instrumentation. However, decentralized tests also have many advantages over Laboratory Developed Tests that are performed at single central laboratories. By avoiding the need for shipping tissues, turnaround times and costs are reduced. The capacity for the laboratory physician to interact directly with the treating physician greatly aids medical care, for example in facilitating appropriate prioritization of critical specimens, explaining equivocal or unexpected results, and quickly recognizing inadequate specimens and what can be done to get a result helpful to the patient as soon as possible. Although first generation breast cancer prognostic tests were performed in central labs [32], second generation tests are being developed and validated to realize the advantages of decentralized testing [39].

The Prosigna assay was tested across a range of RNA mass inputs that is consistent with what will be expected in a clinical lab setting. The assay is robust across that range, similar to what has been reported with other multigene breast cancer tests [32,39]. Additionally, the assay gave consistent results outside the specified assay RNA input limits; only 2 samples failed to produce passing results at half the lowest specified mass further illustrating the robustness of the assay.

The observation of biased subtype calls and ROR scores with the inclusion of non-tumor tissue is consistent with a prior study [40], however the bias reported herein is less severe. Similar to what is expected to be experienced in clinical practice, the interferent being measured here is reported as percent adjacent non-tumor tissue included, rather than percent non-tumor RNA from a separate paired normal tissue sample reported in the earlier study. Normal breast tissue yields less total RNA compared to tumor tissue [41] and adjacent non-tumor tissue at the margins of the tumor have certain cancer pathways activated where matched healthy breast does not [42,43]. Nonetheless, this study illustrates the importance of performing the macrodissection according to the defined procedure to maximize the accuracy of the test.

The precision and reproducibility of the Prosigna assay, estimated from repeat measurements of pooled tumor RNA sample(s) and de-identified patient tissue samples across multiple testing sites is similar (relative to the overall test range) to what was previously reported for centralized lab tests [32,38]. These results demonstrate that the Prosigna assay is analytically reproducible even when performed at multiple test sites and including all process variables. It will be important for local labs to verify the reproducibility reported herein when implementing this decentralized assay to ensure the quality of the test’s results, including ongoing process monitoring [38].

Our experience of implementing the nCounter platform in our CLIA-certified hospital laboratory environments proved to be straightforward, confirming the simplicity of the assay and its suitability as an *in vitro* diagnostic test. Training of the assay workflow (including tissue macrodissection, RNA isolation and setup of Prosigna assay) takes less than one week. The pre-specified SOPs are easy to follow and the procedure of RNA extraction and Prosigna assay are straightforward. All operators, most of whom were naïve users to the Prosigna assay, were able to pass the training procedures on the first attempt, before executing the pre-specified study protocols. Although overnight incubations are required during RNA extraction and RNA – probe hybridization, the incubation temperature is constant, and hands-on time requirements for the whole experiment are very limited. Furthermore, the analyses for subtype call and ROR score are simplified and controlled by integrating the algorithm into the software for raw data processing, reducing the potential for human error in data cleaning and analysis.

## Conclusion

The FDA cleared and CE marked Prosigna assay based on the PAM50 gene expression signature has recently been shown to predict the risk of distant recurrence in women with hormone receptor positive early stage breast cancer treated with five years of endocrine therapy [19,20]. This demonstration of analytical reproducibility generates a strong body of evidence supporting the decentralized use of this test as a tool for breast cancer risk stratification. Additional ongoing studies of the clinical validity of the PAM50 gene expression signature for chemosensitivity prediction [34-36] could, if confirmed, be considered clinically actionable given the demonstrated analytical validity of this test.

## Abbreviations

ROR: Risk of recurrence; SD: Standard deviation; FDA: US Food and Drug Administration; CLSI: Clinical Laboratory and Standards Institute; EGAPP: Evaluation of genomic applications in practice and prevention; FFPE: Formalin-fixed paraffin-embedded.

## Competing interests

TN disclosed that he is one of the holders of the patents on which the Prosigna Assay is based and is a co-founder of Bioclassifier, LLC which licenses the PAM50 algorithm to NanoString Technologies, Inc. JS, SF, BW, ND, and MM all disclose that they are employees of and shareholders in NanoString Technologies. CS is a paid consultant of NanoString Technologies, Inc. All other authors had no disclosures to report.

## Author’s contributions

TN, JS, BW, CS, SF, SL contributed to the study design and protocols and drafted the manuscript. CS, JS, and BW performed statistical analysis and presentation of data. DG performed tissue review for the tissue reproducibility study and the tissue interference study. ND, MM, and GB performed the assay for the RNA precision study. ND and GB performed the assay for the tissue reproducibility study. ND performed the assay for the RNA input and tissue interferents studies. All authors read and approved the final manuscript.

## Pre-publication history

The pre-publication history for this paper can be accessed here:

http://www.biomedcentral.com/1471-2407/14/177/prepub

## Supplementary Material

Additional file 1: Table S1 Site to site ROR sample means. Mean ROR scores were calculated for each pooled RNA sample, and likelihood ratio test for significance was performed to test for differences between sites. There were no significant differences in the results observed across sites for the five pooled RNA samples tested. All p-values were well above 0.05 for the likelihood ratio test of significance of site with 2 degrees of freedom for each pooled RNA sample. The differences in means between sites were all less than 0.5 ROR units on a 0-100 scale. Click here for file

Additional file 2: Table S2 Within site gene expression comparisons from the tissue reproducibility study. Pairwise correlations, slopes, and intercepts of normalized 50 genes for replicate RNA Hybridizations from the tissue reproducibility study. The average intercept, slope, and Pearson’s correlation of the pair-wise comparisons are reported with the 95% confidence interval. Click here for file

Additional file 3: Figure S1 Hierarchical clustering of all samples from the tissue reproducibility study. Clustering analysis (using a Pearson’s distance metric and average linkage) was performed on the median centered normalized, Log2 transformed and scaled sample data to further characterize the gene expression in the tissue samples. The tissue sample and RNA sample replicates were always only clustered together and the node heights are almost imperceptibly low (indicating highly correlated gene expression). Click here for file

Additional file 4: Figure S2 Average ROR Score for the 13 unique tumor RNA samples within the RNA Input Study. Data are colored by the intrinsic subtype result at 250 ng of RNA. Click here for file

Additional file 5: Figure S3 ROR Score for the 23 unique macrodissected tumor samples. Data are colored by the intrinsic subtype result for each tissue. For tissues with multiple isolations the subtype result illustrated was from the macrodissection with the most number of slides processed. Click here for file
