# Supplementary material for: Analytical validation of the PAM50-based Prosigna Breast Cancer Prognostic Gene Signature Assay and nCounter Analysis System using formalin-fixed paraffin-embedded breast tumor specimens
Source: BMC Cancer. 2014 Mar 13;14:177. doi: 10.1186/1471-2407-14-177 (PMC4008304; doi:10.1186/1471-2407-14-177)
Supplement: Additional file 1: Table S1 — Site to site ROR sample means. Mean ROR scores were calculated for each pooled RNA sample, and likelihood ratio test for significance was performed to test for differences between sites. There were no significant differences in the results observed across sites for the five pooled RNA samples tested. All p-values were well above 0.05 for the likelihood ratio test of significance of site with 2 degrees of freedom for each pooled RNA sample. The differences in means between sites were all less than 0.5 ROR units on a 0-100 scale. [file 1471-2407-14-177-S1.doc]

Additional file 1: Tables S1

| Pooled RNA Sample | Site Mean ROR score | | | Likelihood Ratio  Statistic | P-value |
| --- | --- | --- | --- | --- | --- |
|  | 1 | 2 | 3 |  |  |
| Basal-like | 55.5 | 55.4 | 55.3 | 1.14 | 0.566 |
| HER2-enriched | 76.1 | 76.2 | 76.3 | 1.30 | 0.523 |
| Luminal A | 31.3 | 31.4 | 31.3 | 0.43 | 0.808 |
| Luminal B 1 | 54.9 | 55.0 | 55.1 | 1.56 | 0.458 |
| Luminal B 2 | 64.8 | 65.0 | 64.6 | 3.94 | 0.139 |
