# Supplementary material for: Analytical validation of the PAM50-based Prosigna Breast Cancer Prognostic Gene Signature Assay and nCounter Analysis System using formalin-fixed paraffin-embedded breast tumor specimens
Source: BMC Cancer. 2014 Mar 13;14:177. doi: 10.1186/1471-2407-14-177 (PMC4008304; doi:10.1186/1471-2407-14-177)
Supplement: Additional file 2: Table S2 — Within site gene expression comparisons from the tissue reproducibility study. Pairwise correlations, slopes, and intercepts of normalized 50 genes for replicate RNA Hybridizations from the tissue reproducibility study. The average intercept, slope, and Pearson’s correlation of the pair-wise comparisons are reported with the 95% confidence interval. [file 1471-2407-14-177-S2.doc]

Additional file 2: Tables S2

| Comparison | Pairwise Comparisons (n) | Intercept  [95% CI] | Slope  [95% CI] | Pearson  [95% CI] |
| --- | --- | --- | --- | --- |
| All Sites | 124 | 0.00  [-0.01 - 0] | 0.99  [0.99 - 1] | 0.99  [0.99 - 0.99] |
| Site 1 | 40 | -0.01  [-0.01 - 0] | 1.00  [0.99 - 1.01] | 0.99  [0.99 - 0.99] |
| Site 2 | 41 | 0.00  [-0.01 - 0.01] | 0.98  [0.97 - 0.99] | 0.99  [0.99 - 0.99] |
| Site 3 | 43 | 0.00  [-0.01 - 0.01] | 0.99  [0.99 - 1] | 0.99  [0.99 - 0.99] |
