# Supplementary figures and images for: Analytical validation of the PAM50-based Prosigna Breast Cancer Prognostic Gene Signature Assay and nCounter Analysis System using formalin-fixed paraffin-embedded breast tumor specimens
Source: BMC Cancer. 2014 Mar 13;14:177. doi: 10.1186/1471-2407-14-177 (PMC4008304; doi:10.1186/1471-2407-14-177)

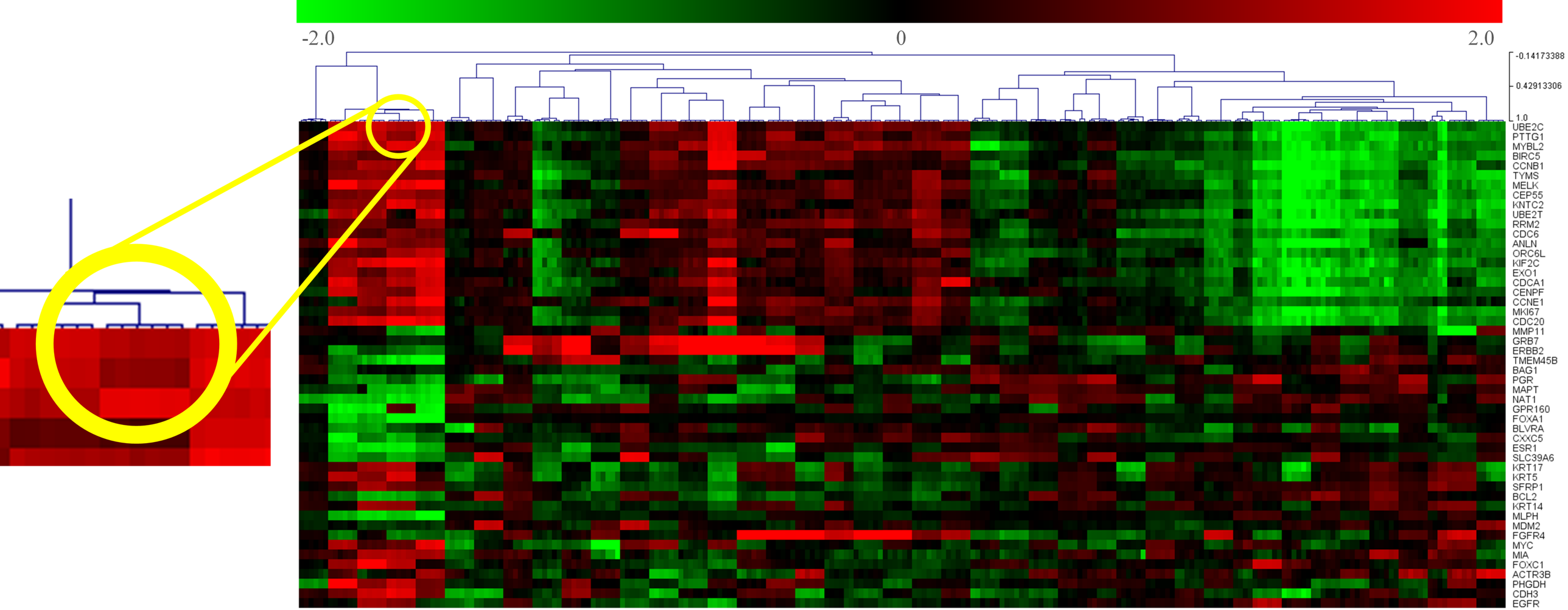

Supplement: Additional file 3: Figure S1 — Hierarchical clustering of all samples from the tissue reproducibility study. Clustering analysis (using a Pearson’s distance metric and average linkage) was performed on the median centered normalized, Log2 transformed and scaled sample data to further characterize the gene expression in the tissue samples. The tissue sample and RNA sample replicates were always only clustered together and the node heights are almost imperceptibly low (indicating highly correlated gene expression). [file 1471-2407-14-177-S3.pdf]

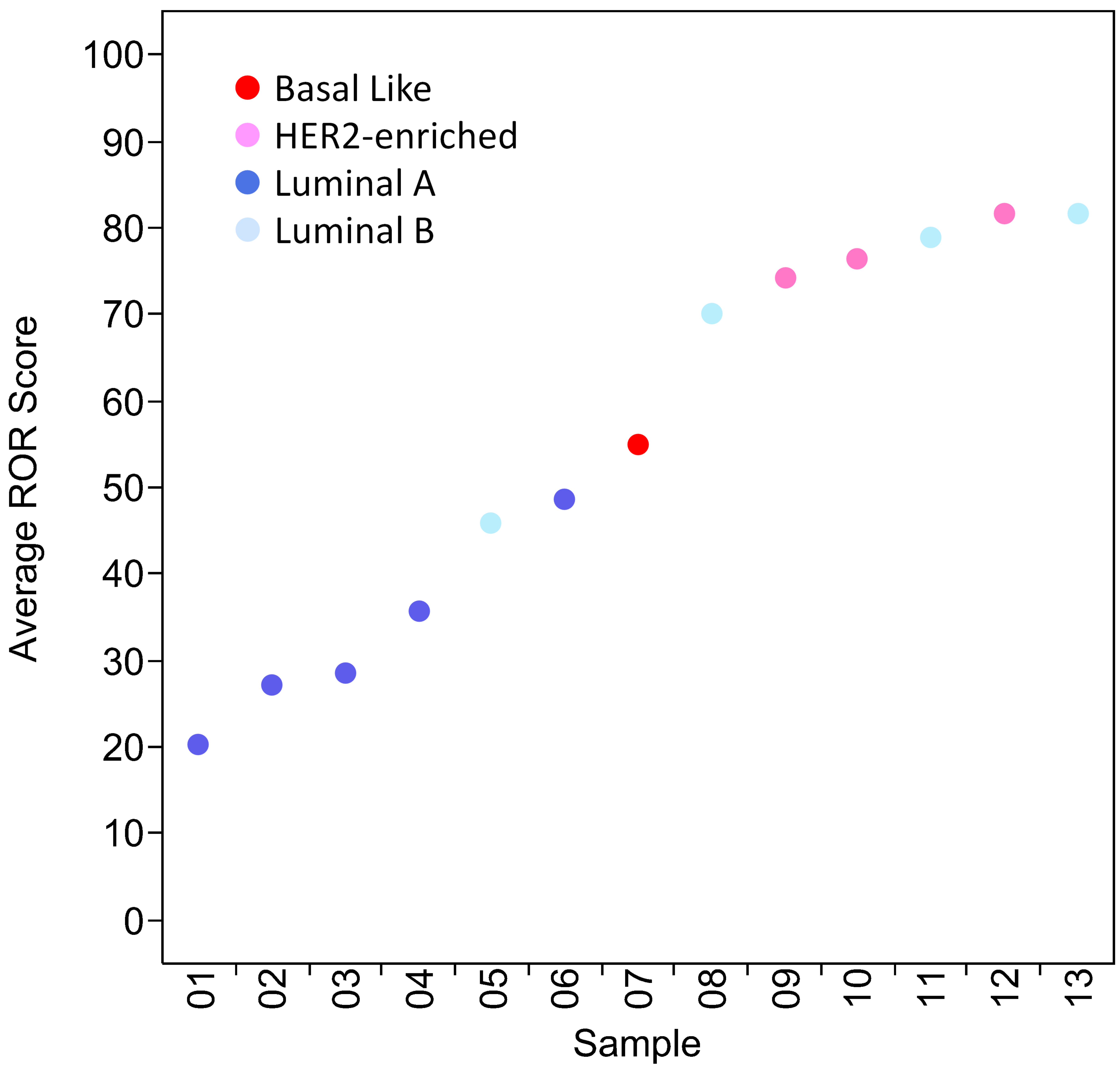

Supplement: Additional file 4: Figure S2 — Average ROR Score for the 13 unique tumor RNA samples within the RNA Input Study. Data are colored by the intrinsic subtype result at 250 ng of RNA. [file 1471-2407-14-177-S4.pdf]

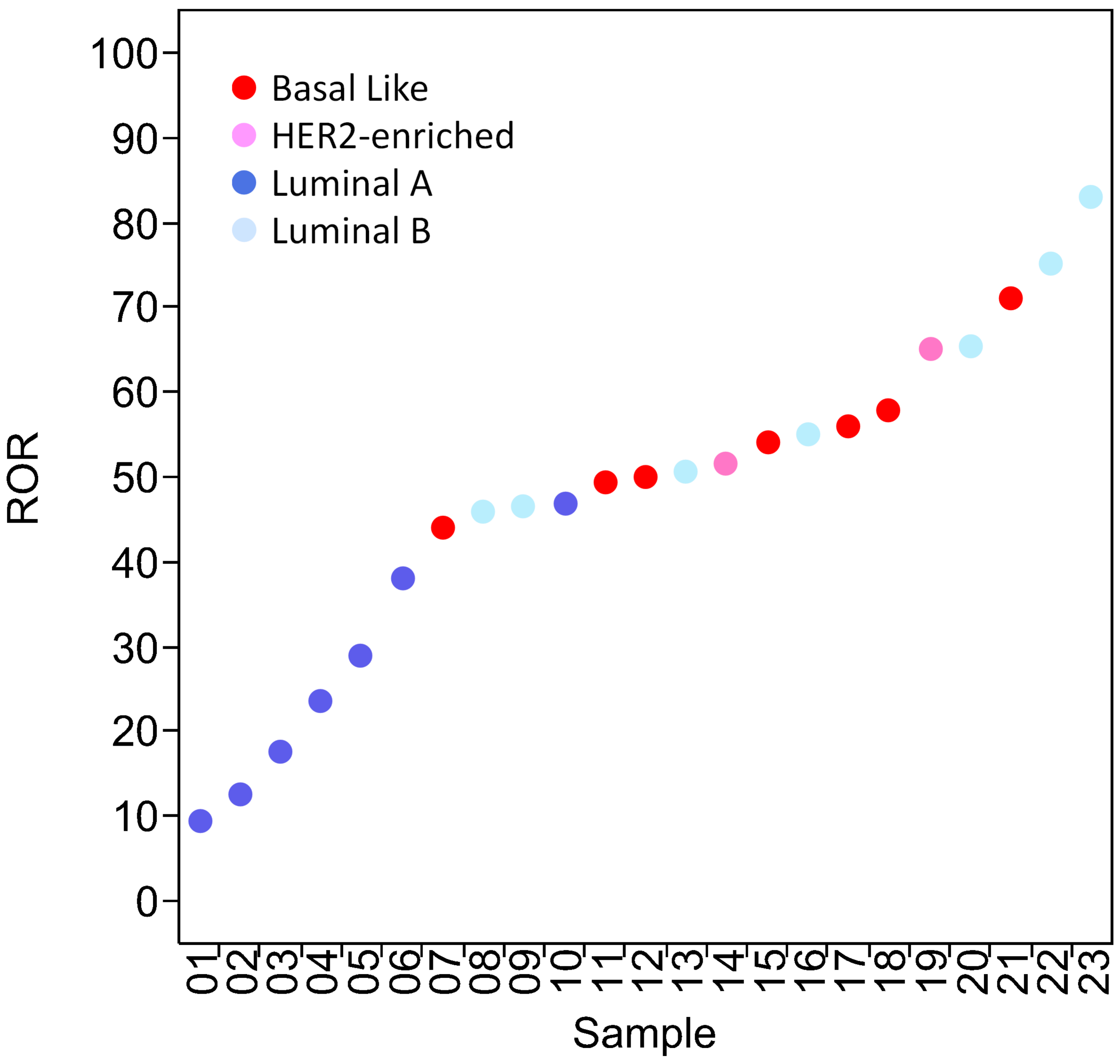

Supplement: Additional file 5: Figure S3 — ROR Score for the 23 unique macrodissected tumor samples. Data are colored by the intrinsic subtype result for each tissue. For tissues with multiple isolations the subtype result illustrated was from the macrodissection with the most number of slides processed. [file 1471-2407-14-177-S5.pdf]
